# Involuntary Psychiatric Holds in Preadolescent Children

**DOI:** 10.5811/westjem.2017.8.35114

**Published:** 2017-09-18

**Authors:** Genevieve Santillanes, Yvette L. Kearl, Chun N. Lam, Ilene A. Claudius

**Affiliations:** University of Southern California, Keck School of Medicine, Department of Emergency Medicine, Los Angeles, California

## Abstract

**Introduction:**

Little is known about the use of involuntary psychiatric holds in preadolescent children. The primary objective was to characterize patients under the age of 10 years on involuntary psychiatric holds.

**Methods:**

This was a two-year retrospective study from April 2013 – April 2015 in one urban pediatric emergency department (ED). Subjects were all children under the age of 10 years who were on an involuntary psychiatric hold at any point during their ED visit. We collected demographic data including age, gender, ethnicity and details about living situation, child protective services involvement and prior mental health treatment, as well as ED disposition.

**Results:**

There were 308 visits by 265 patients in a two-year period. Ninety percent of involuntary psychiatric holds were initiated in the prehospital setting. The following were common characteristics: male (75%), in custody of child protective services (23%), child protective services involvement (42%), and a prior psychiatric hospitalization (32%). Fifty-six percent of visits resulted in discharge from the ED, 42% in transfer to a psychiatric hospital and 1% in admission to the pediatric medical ward. Median length of stay was 4.7 hours for discharged patients and 11.7 hours for patients transferred to psychiatric hospitals.

**Conclusion:**

To our knowledge, this study presents the first characterization of preadolescent children on involuntary psychiatric holds. Ideally, mental health screening and services could be initiated in children with similar high-risk characteristics before escalation results in placement of an involuntary psychiatric hold. Furthermore, given that many patients were discharged from the ED, the current pattern of utilization of involuntary psychiatric holds in young children should be reconsidered.

## INTRODUCTION

Pediatric psychiatric emergencies are increasingly common, even in the very young.^1^–^6^ Large studies have found that more than 20% of visits for pediatric psychiatric emergencies occur in children under the age of 13 years.^7^,^8^ One recent study found that from 2008 to 2015, the percentage of encounters at children’s hospitals for suicidality/self-harm more than doubled with 5–11 year olds accounting for 12.7% of visits.^9^ Despite these findings, little has been published about the nature and severity of psychiatric complaints in young children.

A subset of children seen in emergency departments (ED) for psychiatric complaints are on involuntary psychiatric holds for danger to self or others, or grave disability due to a mental health condition. These involuntary holds are often initiated in the prehospital setting in the midst of a volatile situation, but in reality patients may or may not represent a true and imminent threat to self or others or be gravely disabled. To our knowledge, there is no data published on the use of involuntary psychiatric holds in young children. The effectiveness of involuntary psychiatric holds on stabilizing patients with acute psychiatric emergencies has not been evaluated in adults or adolescents, much less in young children.^10^

The number of patients of any age placed on involuntary psychiatric holds annually in the U.S. is not reliably known.^10^ One might expect the use of involuntary psychiatric holds to be rare in young children because young children rarely have the means to seriously harm themselves or others, and suicide or homicide committed by young children is very uncommon.^11^,^12^ The objectives of this study were to quantify and characterize patients under the age of 10 years on involuntary psychiatric holds seen in one urban public ED in Los Angeles and to determine the ED disposition of these patients.

## METHODS

This was a retrospective study of patients presenting to one ED during a two-year period from April 2013 – April 2015. We conducted the study using established methodology for retrospective chart reviews,^3^,^14^ and included all patients under the age of 10 years (pre-adolescent per the World Health Organization definition) on an involuntary psychiatric hold at any point during their ED visit. Patients were identified by querying the electronic medical record for all patients under the age of 10 with “behavioral precautions,” which is noted for all patients with a recognized psychiatric or behavioral complaint. The University of Southern California Institutional Review Board approved the study with waiver of consent.

### Study Setting

LAC+USC Medical Center is an academic urban county hospital in Los Angeles; it has a dedicated pediatric ED with approximately 24,000 pediatric visits per year. Approximately 1,850 patients under the age of 18 years with psychiatric or behavioral complaints were seen annually during the study period. There are no inpatient psychiatric beds for children on-site, so all children requiring psychiatric inpatient treatment must be transferred to an inpatient psychiatric facility.

In Los Angeles County, children may be placed on an involuntary psychiatric hold by police or parole officers, dedicated psychiatric emergency response teams and designated healthcare providers for grave disability, danger to self or danger to others due to a mental health condition.^15^ The initial hold is valid without judicial review for a period of 72 hours. There is no lower age limit specified in the involuntary psychiatric hold statute for minors. Four psychiatric hospitals in Los Angeles County admit children under the age of 12 years for inpatient treatment.

### Data Collection

We developed a data dictionary prior to initiation of data collection, and data was abstracted by two of the authors, both pediatric emergency attending physicians. The two authors (IC, GS) developed the abstraction protocol together and initial chart abstraction was done with both abstractors present to ensure that abstraction methods were consistent. We collected data using an online system (Survey Gizmo, Boulder, CO) with questions arranged based on the location of data points in the chart. Medical records, including ED records, the involuntary hold, and psychiatric consultations were reviewed. Data collected included the following: basic demographic information; site of hold initiation; reason for hold; living situation; current and prior outpatient psychiatric care; current and prior outpatient psychiatric medication(s); prior psychiatric hospitalizations; and final diagnosis and disposition. For patients admitted to the inpatient pediatric medical service, we collected further details about the reason for admission, admitting diagnoses and laboratory tests. If there was a discrepancy in information in the medical records, we abstracted details of the hold from the legal hold and details of the patient’s history from the psychiatry note. If multiple psychiatry notes existed and had conflicting information, the last child psychiatry note detailing the pertinent information was abstracted.

**Population Health Research Capsule**

What do we already know about this issue?

Pediatric mental health emergencies are commonly seen in the ED and the frequency of these visits is increasing.

What was the research question?

Our goal was to characterize preadolescent patients on involuntary psychiatric holds and report their ED dispositions.

What was the major finding of the study?

Most patients were male, 42% had a history of child protective services contact, and most were discharged home.

How does this improve population health?

The results highlight the need for evaluations of outpatient mental health services and the process of initiation of involuntary psychiatric holds in young children.

### Data Analysis

We used descriptive statistics to characterize the patients. Ten percent of charts were abstracted by both abstractors and a weighted Cohen’s kappa co-efficient was calculated for three variables to measure inter-rater agreement. We conducted statistical tests in STATA 13 (StataCorp, 2013) using two-tailed tests with α set to 0.05.

## RESULTS

We identified 356 patients under the age of 10 years with psychiatric and behavioral complaints. On chart review, we found that 48 patients were not on an involuntary psychiatric hold during their visit and excluded them from the study. These excluded patients were generally patients with co-existing medical and psychiatric diagnoses who were presenting with medical complaints or patients presenting voluntarily for psychiatric medication refills or psychiatric evaluations. A total of 308 visits by 265 unique patients remained for analysis. Of these, 232 patients had one visit during the study period, while 33 patients (12.5%) had repeat visits: 26 patients with two visits, four patients with three visits and three patients with four visits.

Patient characteristics are presented in Table 1. Patients ranged in age from 4–9 years, with 8- and 9-year-old children accounting for 62% of the visits. Of note, 75% of visits were by males. Only 70% were living at home with their parents, and over 40% of the cohort had a known history of child protective services involvement.

At the time of their ED visit 61% of patients were receiving outpatient mental health services, just over half had a history of taking psychiatric medications, and nearly three quarters had received prior mental health treatment. Almost one third of patients reported a prior psychiatric hospitalization. Prior mental health treatment of the study population is presented in Table 2.

Details of the involuntary psychiatric holds are presented in Table 3. The majority of holds were for danger to self or for both danger to self and others. Almost 90% of holds were initiated in the prehospital setting. Holds initiated in the prehospital setting were initiated by police, school police or one of the psychiatric emergency response teams in Los Angeles County.

More than half of patients were discharged home and only 42% were transferred to an inpatient psychiatric facility. The median length of stay (LOS) was 4.7 hours for discharged patients and 11.7 hours for patients transferred to psychiatric hospitals. Further details on disposition are presented in Table 4.

Four patients were admitted to the pediatric medical service. Their median length of stay in the ED was 33.6 hours. One 7-year-old was admitted for observation after a possible overdose of his own medication. An 8-year-old patient was admitted after a 40-hour ED stay in which he was restrained multiple times, refused to eat for over 24 hours and developed mild rhabdomyolysis (creatinine kinase = 1246). Another 7-year-old patient was admitted for mild rhabdomyolysis (creatinine kinase = 2208) after spending more than 24 hours in the ED and having multiple behavioral outbursts. A 9-year-old patient with autism and behavioral problems was admitted to the ward after a nearly four-day ED stay because the father was uncomfortable taking the patient home and no alternate placement could be identified.

When reviewers were compared, the weighted kappa was 0.89 for current psychiatric medications, 0.83 for prior hospitalizations and 0.65 for current living situation.

## DISCUSSION

While involuntary psychiatric holds are a valuable resource in the appropriate setting, they come at a cost both financially and in potential for medical adverse events and psychological repercussions for children and caregivers. Children may be stigmatized, the use of involuntary holds may lead to distrust of social and emergency services by the parents and child, and parents may avoid seeking help in future crisis situations if they feel the hold was not beneficial. Depending on insurance coverage, parents may be responsible for a substantial bill for ambulance transport and ED services. Further research into reasons for placement of involuntary holds, alternative methods of managing behavioral and psychiatric complaints in the prehospital setting and provision of urgent mental health services is warranted.

It would be ideal if alternative methods such as targeted psychiatric screening and improved outpatient resources for at-risk youth could decrease the need for placement of involuntary psychiatric holds. An important trend noted in this study was child protective services involvement. Nearly a quarter of the children in our population were living in a foster home or child protective services temporary congregate care, and over 40% had a known history of child protective services involvement. Children in foster care have a high rate of mental health problems^16^ and account for a disproportionate number of psychiatric hospitalizations.^17^

Children permanently removed from their homes due to child abuse have been shown to be five times more likely than their peers to have an ED visit for suicide-related behavior. 18 Foster children requiring psychiatric inpatient care are more likely to be re-hospitalized,^19^ and children removed from their homes have an increased risk of suicidality and suicide attempts.^20^,^21^ While it is not surprising that a high percentage of preadolescent children on involuntary psychiatric holds in our population were in foster care, this is an accessible population that might benefit greatly from early and frequent mental health and behavioral screening. Additionally, patients with prior psychiatric hospitalizations were highly represented in this cohort. Sixty-one percent of patients in this sample were receiving outpatient mental health care, and 73% had received mental health care in the past. While inpatient hospitalization is sometimes unavoidable, it is possible that more frequent visits, afterhours emergency access, different types of therapy, medications, or other outpatient services could be helpful in preventing acute decompensations.

A re-evaluation of the process by which holds are justified in preadolescent children may be needed. The patients in this sample frequently had mood disorders, adjustment disorders, attention deficit hyperactivity disorder (ADHD) and impulse control disorders. Psychotic disorders were relatively uncommon in this sample. ADHD and impulse control disorder alone are not disorders that typically require inpatient treatment, again raising the question of whether some of these holds were truly indicated.

In this sample, males accounted for 75% of the visits in this cohort of patients. A study of adults on involuntary psychiatric holds documented a slight predominance of men.^22^ Prior literature on the gender of pediatric patients presenting with psychiatric emergencies is mixed with some studies finding a female predominance and others finding a male predominance. 2,5,8,23,24 However, none found such a striking predominance of one gender. It is notable that childhood-onset developmental and psychiatric disorders such as ADHD, autism and conduct disorders show a male predominance, while there is a female predominance in mood and anxiety disorders, which more commonly present in adolescence.^25^ Boys tend to display more externalizing symptoms,^26^ which may contribute to the placement of involuntary psychiatric holds. This population was replete with stories of young boys acting out or drawing battles, and being placed on involuntary holds. One child, in particular, was directly quoting a popular children’s television series, resulting in an involuntary hold for “danger to others.”

Laws regarding involuntary psychiatric hospitalization vary greatly from state to state.^10^ In Los Angeles County, 72-hour involuntary psychiatric holds may be initiated by a variety of professionals including police and parole officers and psychiatric emergency response teams.^15^ In our patient population, psychiatric holds were most frequently placed by police, school police and psychiatric emergency response teams. In Los Angeles County, there are many separate police agencies and psychiatric emergency response teams, making uniform training and application of hold criteria challenging. The training and comfort level with psychiatric emergencies in general and especially pediatric psychiatric emergencies likely varies greatly by type of responder. It is possible that psychiatric emergency response teams with specialized training in pediatric behavioral, developmental and mental health would be able to de-escalate more emergency calls without requiring placement of an involuntary psychiatric hold.

The final question that arises is whether the child ultimately benefited from the hold placement. While not directly addressed in this study, the median ED LOS for discharged patients was 4.7 hours. While this LOS does represent a burden on ED resources, it is hard to imagine that meaningful psychiatric stabilization and treatment could occur in such a short time period. Prior data would indicate that boarding is non-therapeutic for the majority of psychiatric patients.^27^ This relatively short LOS and the fact that over half of the involuntary holds were overturned in the ED raises the question of whether these holds were truly necessary. It is possible that in some cases involuntary holds could be avoided if more robust urgent outpatient services were available so that prehospital psychiatric response teams could instead link patients and families to appropriate services rather than placing a hold.

Clearly, further study is necessary to assess the benefit of targeted screening and referral in preventing involuntary holds. Involuntary hold criteria may need to differ by age and developmental level. Young children have lower rates of suicide and homicide and can generally be supervised by parents or other caretakers. Given these differences from an adult population, if intensive outpatient services were available urgently for children in crisis, perhaps this would be a better alternative, particularly for young children. This may be especially true for the patients already receiving outpatient care as their caretakers have shown a willingness to seek mental health treatment for their child. Significant gaps in outpatient mental health resources for children have been documented, due in part to reimbursement issues.^28^ Investment in pediatric outpatient mental health services would likely be beneficial to patients and might decrease the need for costly ED and inpatient services resulting from psychiatric and behavioral crises leading to involuntary psychiatric holds.

## LIMITATIONS

Although we generally followed the methodologies for conducting a retrospective review outlined by Kaji et al. and Gearing et al.,^13^,^14^ the abstractors were study investigators. We felt that blinding of the abstractors to the study hypothesis was not necessary since this was purely a descriptive study of characteristics of the population and detailed abstraction protocols were developed a priori.

This study is limited by the biases of a retrospective chart review. Ideally, we would have compared this population of patients to patients presenting with non-psychiatric complaints. However, as detailed social histories are not generally documented on patients presenting with non-psychiatric complaints, this would have clearly introduced bias in favor of the identified characteristics. Even in the patients presenting with psychiatric and behavioral complaints, historical details were not always immediately available and therefore may not have been documented. For example, the reason for prior hospitalization or outpatient treatment was not reliably documented. The 33 patients with a documented repeat visit represent only patients who had a repeat visit to our ED during the study period and before the age of 10. This number almost certainly underestimates recidivism, as patients with subsequent involuntary psychiatric holds would have been missed if their subsequent visit occurred after the study period or in another ED or psychiatric inpatient facility.

Hispanic patients comprised 56% of the visits in this sample, which is close to the expected based on the demographics of Los Angeles County. At the time of the 2010 census, 47.7% of the county’s population was of Hispanic or Latino origin.^29^ The hospital serves an overwhelmingly Hispanic/Latino population, with approximately 70% of the patients being of Hispanic/Latino descent. This data represents the patients seen in one urban county ED with a high proportion of Hispanic/Latino patients and may not be representative of other populations.

## CONCLUSION

We have presented the first characterization of preadolescent children on involuntary psychiatric holds, many of whom were ultimately managed as outpatients. Given the potential for harm, the lack of proven benefit,^10^ and the fact that most holds in this population were overturned, the current pattern of utilization of involuntary psychiatric holds in young children should be reconsidered. Further research is needed to identify effective means of proactively providing services to avoid the need for involuntary psychiatric holds, ED visits and short-term emergency hospitalizations. In particular, foster children and those who have had contact with child protective services or the inpatient mental health system in the past might benefit from aggressive screening and intervention if mental health issues are identified.

## Figures and Tables

**Table 1:** Characteristics of preadolescent patients by visit to the emergency department (ED) for psychiatric and behavioral complaints (N=308).

	N	%
Age (years)		
4	3	1.0
5	23	7.5
6	39	12.7
7	53	17.2
8	82	26.6
9	108	35.1
Sex		
Male	231	75.0
Female	77	25.0
Race/ethnicity		
Hispanic/Latino	173	56.2
African-American	78	25.3
White, non-Hispanic	34	11.0
Asian	6	1.9
Other	9	2.9
Unknown	8	2.6
Living situation		
Home	215	69.8
Foster home	55	17.9
Temporary congregate care (child protective services)	15	4.9
Group home	9	2.9
Other	13	4.2
Unknown	1	0.3
Known current or prior child protective services involvement	128	41.6
New child protective services report made in ED	25	8.1

**Table 2:** Prior mental health treatment of preadolescent patients by visit (N=308).

	Yes; N (%)	No; N (%)	Unk; N (%)
In outpatient treatment at time of visit	189 (61.4)	103 (33.4)	16 (5.2)
On psychiatric medications at time of visit	140 (45.5)	162 (52.6)	6 (1.9)
Any history of psychiatric care (inpatient or outpatient)	225 (73.1)	70 (22.7)	13 (4.2)
Any history of psychiatric medications (current or past)	161 (52.3)	129 (41.9)	18 (5.8)
Prior psychiatric hospitalization	100 (32.5)	190 (61.7)	18 (5.8)

**Table 3:** Details of involuntary psychiatric holds (N=308).

	N	%
Reason for hold		
Danger to self	112	36.4
Danger to others	51	16.6
Danger to self and others	131	42.5
Gravely disabled (including co-diagnosis)	6	1.9
Unknown	8	2.6
Setting where hold initiated		
Prehospital	276	89.6
LAC+USC psychiatric outpatient clinic	4	1.3
LAC+USC emergency department	27	8.7
Unknown	1	0.3

**Table 4:** Disposition of visits (N=308).

	N	%
Disposition		
Discharged	174	56.5
Transferred to a psychiatric hospital	130	42.2
Admitted to the pediatric medical ward	4	1.3
LOS by disposition, in hours	Median	Range
Discharged	4.7	1.09 – 95.25
Transferred to a psychiatric hospital	11.7	0.4 – 243.25
Admitted to the pediatric medical ward	33.6	7.21 – 93.1

*LOS*, length of stay.
